# The novel 19q13 KRAB zinc-finger tumour suppressor ZNF382 is frequently methylated in oesophageal squamous cell carcinoma and antagonises Wnt/β-catenin signalling

**DOI:** 10.1038/s41419-018-0604-z

**Published:** 2018-05-14

**Authors:** Chong Zhang, Tingxiu Xiang, Shuman Li, Lin Ye, Yixiao Feng, Lijiao Pei, Lili Li, Xiangyu Wang, Ran Sun, Guosheng Ren, Qian Tao

**Affiliations:** 1 Chongqing Key Laboratory of Molecular Oncology and Epigenetics, The First Affiliated Hospital of Chongqing Medical University, Chongqing, China; 2 Department of Oncology, The Second Affiliated Hospital of Chongqing Medical University, Chongqing, China; 3 Cancer Epigenetics Laboratory, Department of Clinical Oncology, State Key Laboratory of Oncology in South China, Sir YK Pao Center for Cancer and Li Ka Shing Institute of Health Sciences, The Chinese University of Hong Kong and CUHK-Shenzhen Research Institute, Hong Kong, Hong Kong

## Abstract

Zinc finger proteins (ZFPs) are the largest transcription factor family in mammals. About one-third of ZFPs are Krüppel-associated box domain (KRAB)-ZFPs and involved in the regulation of cell differentiation/proliferation/apoptosis and neoplastic transformation. We recently identified *ZNF382* as a novel KRAB-ZFP epigenetically inactivated in multiple cancers due to frequent promoter CpG methylation. However, its epigenetic alterations, biological functions/mechanism and clinical significance in oesophageal squamous cell carcinoma (ESCC) are still unknown. Here, we demonstrate that *ZNF382* expression was suppressed in ESCC due to aberrant promoter methylation, but highly expressed in normal oesophagus tissues. *ZNF382* promoter methylation is correlated with ESCC differentiation levels. Restoration of *ZNF382* expression in silenced ESCC cells suppressed tumour cell proliferation and metastasis through inducing cell apoptosis. Importantly, *ZNF382* suppressed Wnt/β-catenin signalling and downstream target gene expression, likely through binding directly to *FZD1* and *DVL2* promoters. In summary, our findings demonstrate that *ZNF382* functions as a bona fide tumour suppressor inhibiting ESCC pathogenesis through inhibiting the Wnt/β-catenin signalling pathway.

## Introduction

Oesophageal cancer is the eighth most common cancer with the sixth highest cancer mortality rate worldwide and a very low 5-year survival of 15%–25%^1,2^. Oesophageal squamous cell carcinoma (ESCC) comprises 90% of oesophageal cancer as the predominant type in China; the remaining cases are oesophageal adenocarcinoma (EAC)^1,3^. The incidence of oesophageal cancer varies geographically with the highest in a belt extending through central Asian to North-Central China^1,2,4,5^, reaching an incidence of >100/100,000 population annually^2,6,7^. The major risk factors for ESCC are poor nutrition, tobacco and alcohol consumption, whereas diet, obesity and gastroesophageal reflux disease are also EAC risk factors^4,8,9^. The incidence of ESCC has been decreasing a bit, but its 5-year survival remains poor; therefore a better understanding of ESCC pathogenesis is urgently needed^3,6,10–13^. Substantial evidences have shown that epigenetic modifications, primarily aberrant promoter CpG methylation of tumour suppressor genes (TSGs), consistently contribute to malignant transformation^4,14,15^. Alterations of promoter hypermethylation occur frequently in ESCC and involve multiple genes required for ESCC carcinogenesis^14,16^. Therefore, it is crucial to investigate the epigenetic abnormalities in ESCC.

Zinc finger proteins (ZFPs) comprise the largest group of transcription factors. Their zinc finger domains bind to gene promoters to activate or repress gene expression^17^. Nearly one-third of mammalian ZFPs contain a highly conserved Krüppel-associated box (KRAB) motif, which contributes to transcriptional repression by recruitment of histone deacetylase (HDAC) complexes^18–21^. We recently identified a novel ZFP *ZNF382*, the human homologue of rat tumour suppressor KS1 located at 19q13.12, that functions as a transcriptional repressor by binding to the KAP-1 co-repressor protein^22^. *ZNF382* is widely expressed in normal tissues but reduced or silenced in multiple carcinomas due to aberrant promoter CpG methylation^22–24^. Although evidence has indicated that ectopic expression of *ZNF382* suppresses tumour cell proliferation and promotes apoptosis, its biological functions and underlying mechanisms in ESCC pathogenesis remain to be investigated^24^.

Wnt signalling includes canonical Wnt/β-catenin pathway and noncanonical pathway and is an oncogenic activation event in many cancers, especially in gastrointestinal cancers. Numerous studies have reported that Wnt signalling is associated with the initiation and progression of human ESCC. Furthermore, comprehensive genomic analysis of ESCC revealed that altered genes in Wnt pathway were identified in ~86% of ESCC cases, adding to our understanding of pathogenenic role of Wnt pathway in ESCC tumorigenesis^25,26^. In this study, we investigated the methylation status of *ZNF382* in primary ESCC and its biological functions in silenced ESCC cell lines. We further explored the mechanism of *ZNF382* on tumour suppression of ESCC.

## Results

### Promoter methylation leads to ZNF382 downregulation in ESCC

**Fig. 1:**
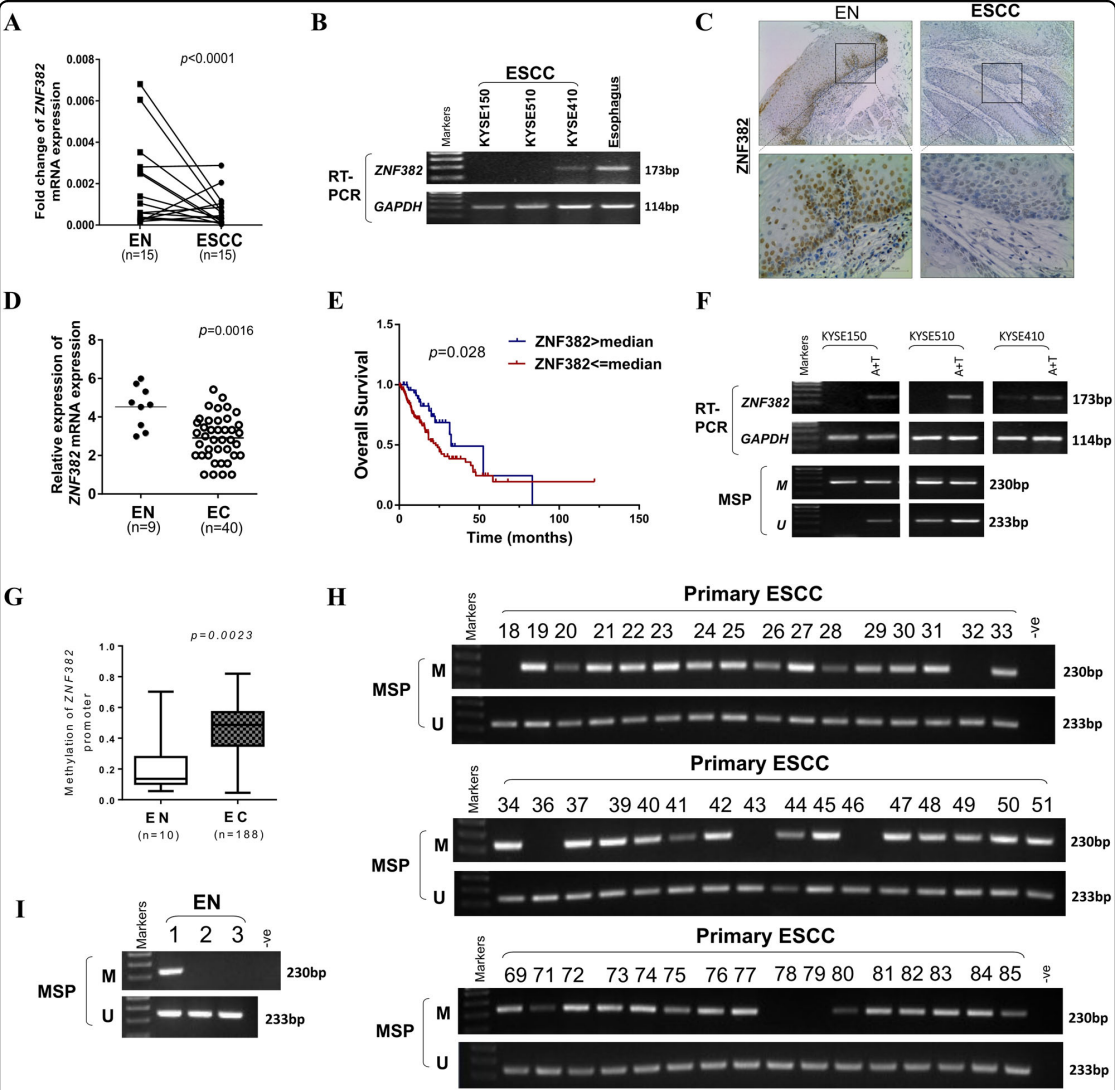
ZNF382 downregulation in ESCC due to frequent promoter CpG methylation. **a** *ZNF382* expression in primary ESCC (*n* = 15) and paired adjacent non-cancerous tissues (*n* = 15) by qRT-PCR. Mean ± SD, *p* *<* 0.001. **b** *ZNF382* expression in representative ESCC cells compared with normal oesophageal tissues was assayed by RT-PCR. **c** Representative IHC for ZNF382 in ESCC tissues (right) and paired adjacent non-cancerous tissues (left). **d** *ZNF382* expression in ESCC tissues (*n* = 40) and normal oesophageal tissues (*n* = 9) from online Gene Expression across Normal and Tumour Tissues (GENT) database (http://medical-genome.kribb.re.kr/GENT/search/search.php). **e** Kaplan–Meier cumulative survival curves of *ZNF382* expression in oesophageal cancer patients are presented to explore the prognostic implication of *ZNF382*. Data were extracted from the Cancer Genome Analysis Database, and *p* value indicated statistical significance. **f** Restoration of *ZNF382* expression with 5-aza-2′-deoxycytidine (Aza) and trichostatin A (TSA) treatments in ESCC cells. Demethylation was measured by MSP. M methylated, U unmethylated. **g** Methylation status of *ZNF382* in oesophageal cancer tissues (*n* = 188) and normal oesophagus tissues (*n* = 10) from human disease methylation database (http://www.biobigdata.com/diseasemeth/). **h**–**l** *ZNF382* methylation in primary ESCC tissues (*n* = 114) and normal tissues (*n* = 3), measured by methylation-specific PCR (MSP). M methylated, U unmethylated

*ZNF382* downregulation in some carcinomas has been previously reported^24^. We thus further analysed *ZNF382* mRNA expression via qRT-PCR in 15 cases of ESCC and paired adjacent non-cancerous tissues, and we found that *ZNF382* expression in the ESCC samples was significantly reduced compared with paired adjacent non-cancerous tissues (Fig. 1a). We then evaluated *ZNF382* expression in a panel of ESCC cell lines and normal oesophagus tissues by real-time PCR (Fig. 1b) and qRT-PCR (Supplemental Fig. 1). *ZNF382* was highly expressed in normal oesophagus tissues, but almost completely silenced in KYSE150, KYSE410 and KYSE510 cells. We also examined *ZNF382* protein expression in ESCC and paired adjacent non-cancer tissues by immunohistochemistry (IHC). *ZNF382* protein expression was significantly weaker in ESCC than in adjacent non-cancerous tissues and primarily localised in cell nuclei (Fig. 1c). In addition, *ZNF382* expression was analysed using the online Gene Expression across Normal and Tumour (GENT) tissue database (http://medical-genome.kribb.re.kr/GENT/search/search.php), and *ZNF382* was also found to be downregulated in ESCC tissues compared with normal oesophageal epithelial tissues (Fig. 1d, *p*= 0.0016). Furthermore, the correlation of *ZNF382* expression and overall survival (OS) in oesophageal cancer patients was investigated according to the cBioPortal for Cancer Genomics (http://www.cbioportal.org/) in The Cancer Genome Atlas (TCGA) database. Higher levels of *ZNF382* expression were associated with a better OS rate in oesophageal cancer patients (Fig. 1e, *p=* 0.028), suggesting that *ZNF382* may be an independent prognostic factor in oesophageal cancer.

Abnormal promoter methylation is a critical mechanism responsible for the silencing of TSGs in tumourigenesis^27^. Our previous research demonstrated that *ZNF382* is frequently methylated in solid cancers. To observe whether *ZNF382* expression is regulated by promoter methylation, we treated KYSE150, KYSE410 and KYSE510 cells with the demethylation drug Aza combined with the HDAC inhibitor trichostatin A (TSA), and the synergistic treatments significantly restored *ZNF382* expression, together with increased unmethylated alleles and decreased methylated alleles (Fig. 1f). In addition, *ZNF382* promoter methylation in primary oesophageal cancer tissues and normal oesophagus tissues from a human disease methylation database (http://www.biobigdata.com/diseasemeth/) was analysed. *ZNF382* methylation was far more prevalent in oesophageal cancer tissues than in normal oesophagus tissues (Fig. 1g, *p=* 0.0023). Thus, the overall results indicate that promoter methylation may be a primary mechanism of *ZNF382* silencing in ESCC cell lines.

### ZNF382 methylation in ESCC is correlated with clinicopathological features

To further investigate *ZNF382* promoter methylation status in ESCC, methylation-specific PCR (MSP) was performed in primary ESCC and the normal oesophagus tissues. The data showed that the *ZNF382* promoter was hypermethylated in 87% (99/114) of ESCC tissues and in 33% (1/3) of normal oesophageal tissues, suggesting the frequent methylation of *ZNF382* in ESCC (Fig. 1h–i and Table 1). Next, we analysed the relationship between *ZNF382* methylation status and clinical features, and a significant association was found with tumour differentiation (Table 2) (χ^2^ test, *p=* 0.029).

**Table 1:** The promoter methylation of ZNF382 in primary ESCC

Samples	ZNF382 methylation status		Frequency of **methylation**
	**Methylated**	**Unmethylated**	
ESCC (*n* = 114)	99	15	87% (99/114)
EN (*n* = 3)	1	2	33% (1/3)
Adjacent tissues (n=16)	12	4	75%(12/16)

*ESCC* Oesophageal squamous cell carcinoma, *EN* Normal oesophagus tissues

**Table 2:** The correlation between *ZNF382* promoter methylation and clinicopathological feature in ESCC

Clinicopathological features	Numbers	ZNF382 methylation status		
	*N* = (114)	Methylated	Unmethylated	*p* value
Age				*0.422*
≤ 60	35	30	5	
> 60	79	68	11	
Gender				*0.366*
Male	92	78	14	
Female	22	20	2	
Tumour Location				*0.411*
Upper	17	14	3	
Middle	56	47	9	
Lower	40	37	3	
Unknown	1	0	1	
Differentiation				***0.029***
Moderately/Well	89	80	9	
Poorly	25	18	7	
Tumour Grade				*0.076*
T1–2	34	30	4	
T3–4	79	68	11	
Unknown	1	0	1	
Tumour Size				*0.465*
>5.0 cm	19	17	2	
≥2.0 cm ≤5.0 cm	75	67	8	
<2.0 cm	14	12	2	
Unknown	6	4	2	
Tumour Stage				*0.089*
I/II	70	61	9	
III/IV	43	37	6	
Unknown	1	1	0	
Lymph Nodes Metastasis				*0.356*
Positive	40	34	6	
Negative	74	65	9	
Distant Metastasis				*0.336*
Positive	1	1	0	
Negative	113	97	16	

The presence of italics values means whether there is a significant association between ZNF382 methylation and the feature. As p<0.05 was set to be statistically significant, so p=0.029 means there's statistically significant association between ZNF382 methylation and tumor differentiation

### Ectopic expression of ZNF382 suppresses ESCC cell proliferation

**Fig. 2:**
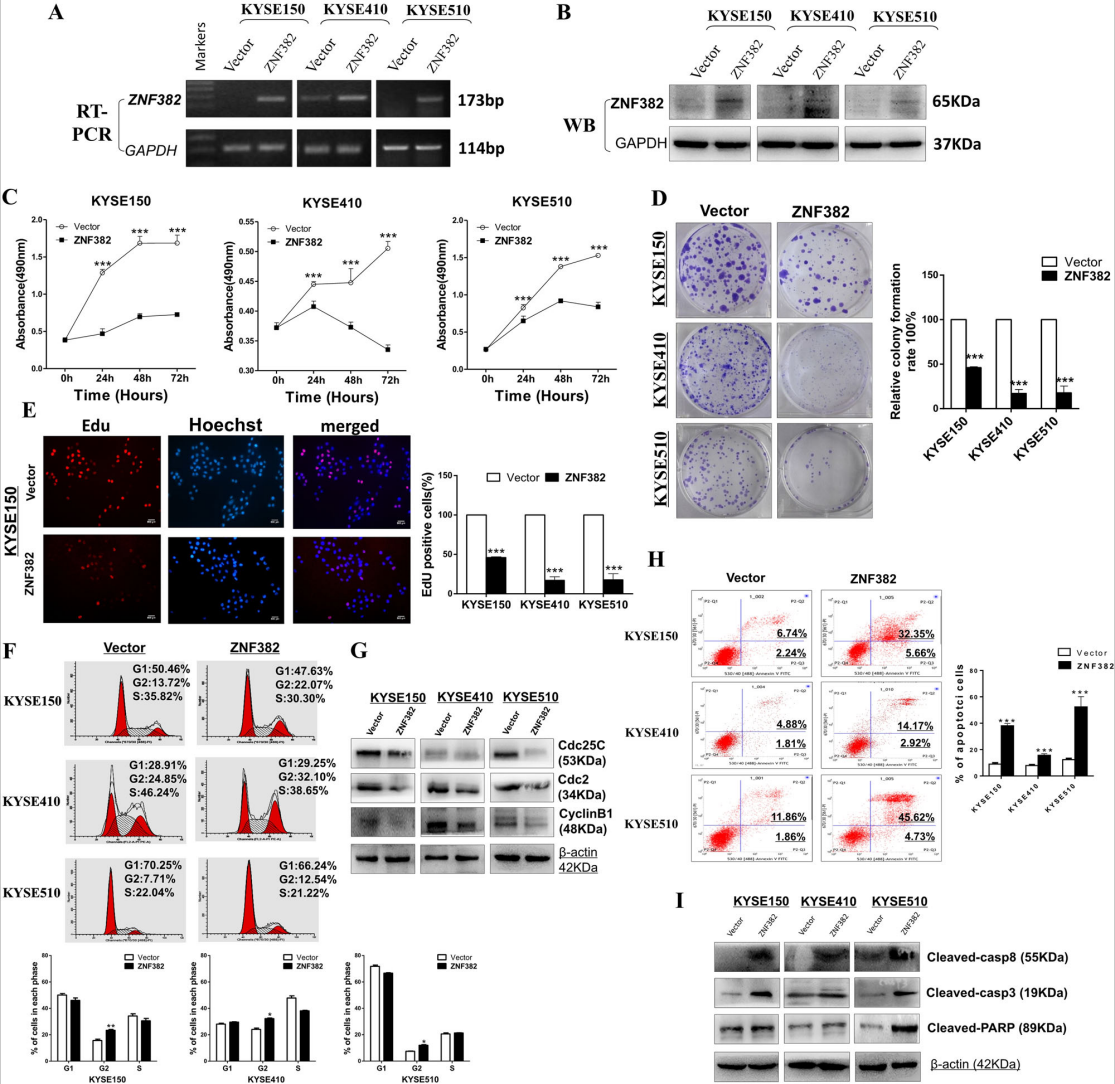
ZNF382 suppresses proliferation and induces G2/M cell cycle arrest and apoptosis in ESCC cells. **a**, **b** Ectopic expression of *ZNF382* in ESCC cells was measured by RT-PCR and western blot assays, respectively. **c** Cell viabilities were evaluated at 24, 48 and 72 h after transfection with *ZNF382* in KYSE150, KYSE410 and KYSE510 cells. Mean ± SD, ***p *<* 0.001. **d** Representative images of showing the colony formation ability of in vector- and of *ZNF382*- transfected KYSE150, KYSE410 and KYSE510 cells (left panel). The colony formation rates are shown as the mean ± SD (right panel) from three independent experiments (***p< 0.001). **e** Edu cell proliferation assays of KYSE150, KYSE410 and KYSE510 cells. Scale bars: 800 μm. Data are from three independent assays and are shown as the mean ± SD, ***p *<* 0.001. **f** The cell cycle distribution was measured in vector- and ZNF382-stably transfected KYSE150, KYSE410 and KYSE510 cells by flow cytometry analysis. Representative cell cycle distribution plots and histograms of cell cycle alterations are shown (**p <0.01, *p< 0.05). **g** Western blot assay of Cdc25C, Cdc2 and CyclinB1 protein expression levels. **h** The percentages of apoptotic cells in KYSE150, KYSE410 and KYSE510 cells with *ZNF382* ectopic expression were evaluated. Cell apoptosis alterations were revealed by histograms (***p < 0.001). **i** The expression of cleaved-caspase8, cleaved-caspase3 and cleaved PARP was measured by western blot assay in *ZNF382*-transfected ESCC cells. All experiments were repeated in triplicate and are shown as the mean ± SD

*ZNF382* ectopic expression in *ZNF382*-stably transfected KYSE150, KYSE410 and KYSE510 cells was verified by RT-PCR and western blot, respectively (Fig. 2a, b). To investigate the effect of *ZNF382* on ESCC cell proliferation, cell viability and colony formation assays were performed. *ZNF382*-transfected cells exhibited lower viability at 24, 48 and 72 h (Fig. 2c) and 60% to 80% less colony formation than controls (Fig. 2d). In addition, we also carried out Edu cell proliferation assays, and a significant decrease in the percentage of Edu-positive cells was observed after transfection of ESCC cells with *ZNF382* (Fig. 2e and Supplemental Figure 2).

### ZNF382 induces G2/M cell cycle arrest and apoptosis in ESCC cells

A cell cycle assay was performed to determine how *ZNF382* affects cell proliferation. Compared with controls, *ZNF382* significantly increased KYSE150, KYSE410 and KYSE510 cells in the G2/M phase after *ZNF382* transfections (Fig. 2f). This effect was accompanied by Cdc25C, Cdc2 and CyclinB1 inhibitions (Fig. 2g). Flow cytometry analysis of cell apoptosis was performed to evaluate the mechanisms of *ZNF382* suppression of tumour cell growth. As shown in Figure 2h, the percentage of apoptotic cells was remarkably increased after *ZNF382*- transfection in ESCC cells. Caspases were involved in the proteolytic cascade, which further induced apoptosis. Western blots showed that *ZNF382* significantly increased the cleavage of caspase8, caspase3 and PARP in ESCC cells compared with vector controls (Fig. 2i).

### ZNF382 inhibits ESCC cell migration and invasion

**Fig. 3:**
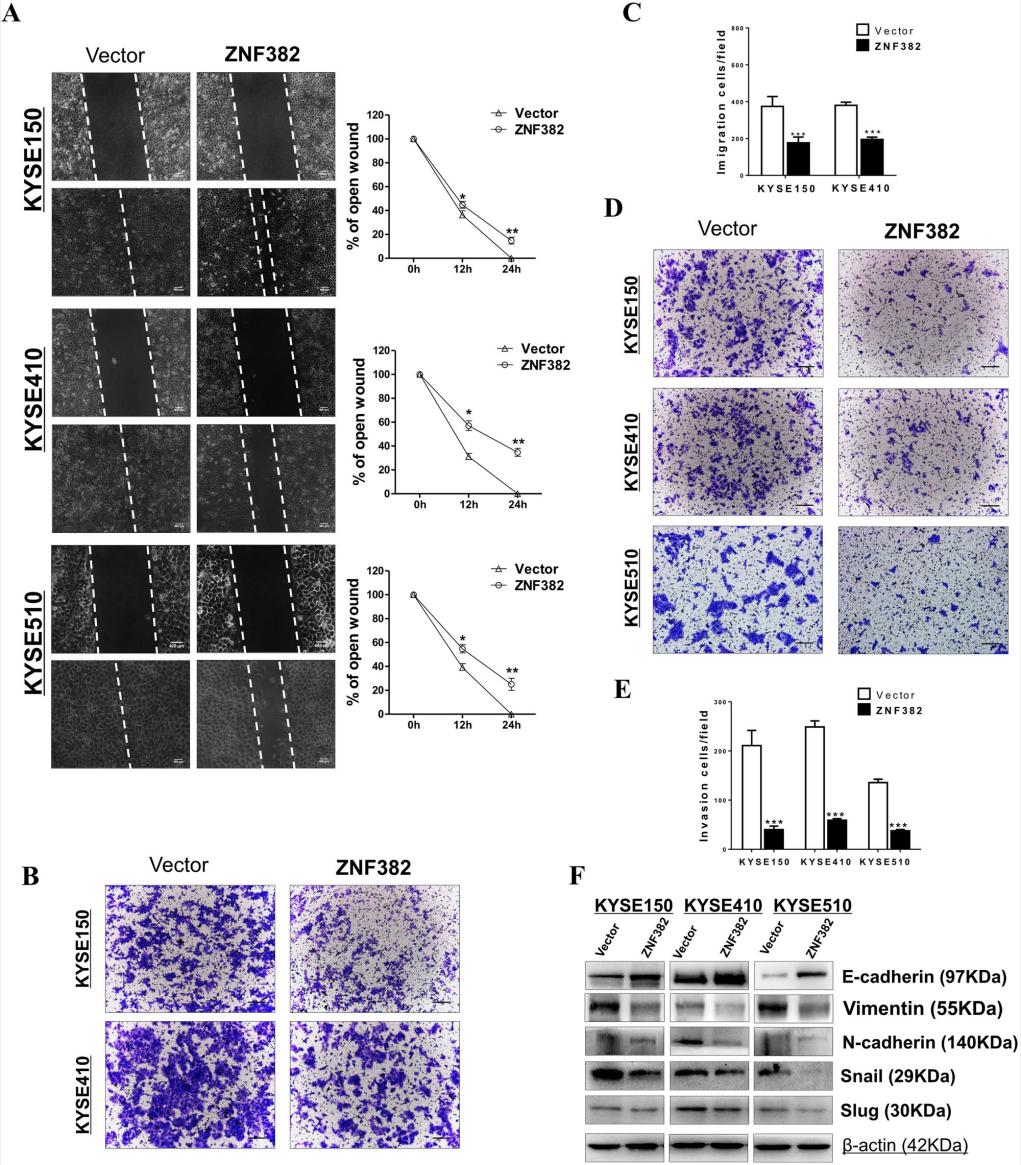
ZNF382 inhibits cell migration and invasion of ESCC cells. **a** The cell migration abilities of ESCC cells were evaluated by wound healing assays. Photographs were captured at 0, 12 and 24 h. The representative wound healing ratio is shown. Scale bars: 400 μm. Mean ± SD, **p *<* 0.01, *p *<* 0.05. The cellular migration (**b**, **c**) and invasion (**d**, **e**) abilities of ESCC cells upon ectopic expression of *ZNF382* were measured by transwell assays with or without Matrigel. Representative images were photographed following fixation and staining. Assays were performed in triplicate, ***p< 0.001. Scale bars: 100 μm. **f** Expression of representative EMT markers by western blot assay

To investigate the effect of *ZNF382* on cell migration, wound healing assays were carried out. *ZNF382-*stably transfected ESCC cells migrated into the wounded areas slower than the control cells at 24 h (Fig. 3a). Moreover, the effect of *ZNF382* on cell migration and invasion were further analysed by transwell assay. The results showed that ectopic expression of *ZNF382* significantly decreased the number of migrating (Fig. 3b, c) and invading cells (Fig. 3d, e) compared with controls. The effect of *ZNF382* on ESCC cell metastasis was mediated by E-cadherin (CDH1) upregulation and vimentin (VIM), N-cadherin (CDH2), SNAIL1 and SLUG downregulation (Fig. 3f).

### Knockdown of ZNF382 promotes cell growth and induces metastasis

**Fig. 4:**
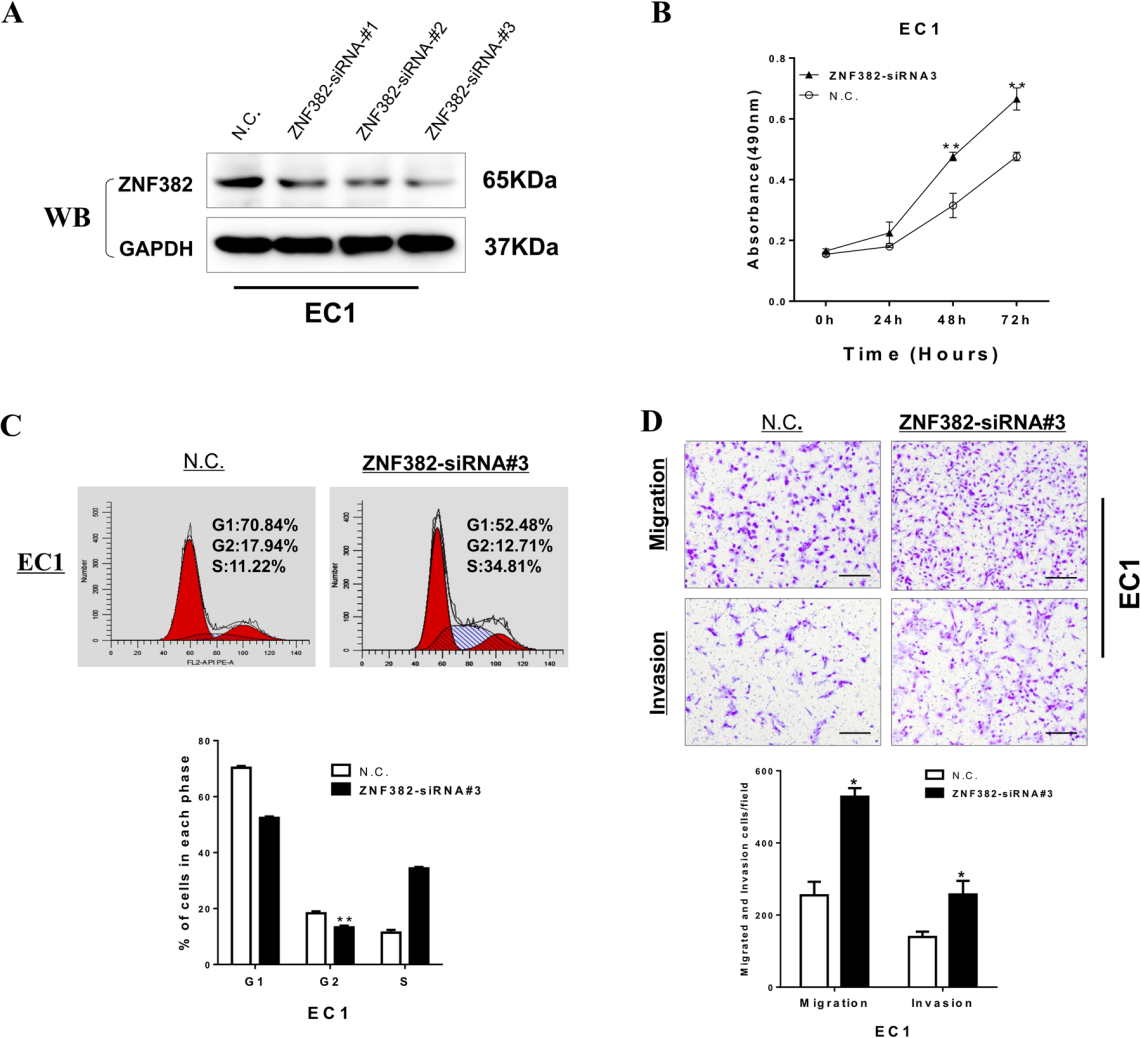
Knockdown of ZNF382 promotes ESCC cell growth and induces metastasis. **a** Evaluation of *ZNF382* knockdown in EC1 cells after transfection with *ZNF382* siRNA and si-Scramble by western blot. **b** Knockdown of *ZNF382* on cell viability was measured by cell viability assay. Each experiment was repeated three times. **p <0.01. **c** Cell cycle distribution after *ZNF382* knockdown in EC1 cells. Each experiment was repeated three times. **p <0.01. **d** Transwell assay showed cell migration and invasion of EC1 cells after *ZNF382* knockdown. Scale bars: 200 μm. Each experiment was repeated three times. *p< 0.05

To further validate the suppression function of *ZNF382* in ESCC, we investigated the effect of *ZNF382* through knockdown of *ZNF382* in *ZNF382*-expressing ESCC cell line EC1 by siRNA transfection. Knockdown efficiency was detected by western blot (Fig. 4a). Knockdown of *ZNF382* markedly increased EC1 cell viability, according to CCK8 assays compared with cells transfected with control siRNA (Fig. 4b). To evaluate the effect of *ZNF382* on cell cycle regulation, flow cytometry assays were employed. The distribution of EC1 cells in G2/M phase was significantly reduced after knocking down *ZNF382* (Fig. 4c). Moreover, we observed enhanced cell migration and invasion after *ZNF382* knocking down. Compared with control, the number of migrated and invading EC1 cells were dramatically increased after knockdown of *ZNF382* (Fig. 4d). These knockdown data confirmed that *ZNF382* functions as a tumour suppressor in ESCC cells.

### Identification of differentially expressed genes regulated by ZNF382 in ESCC

**Fig. 5:**
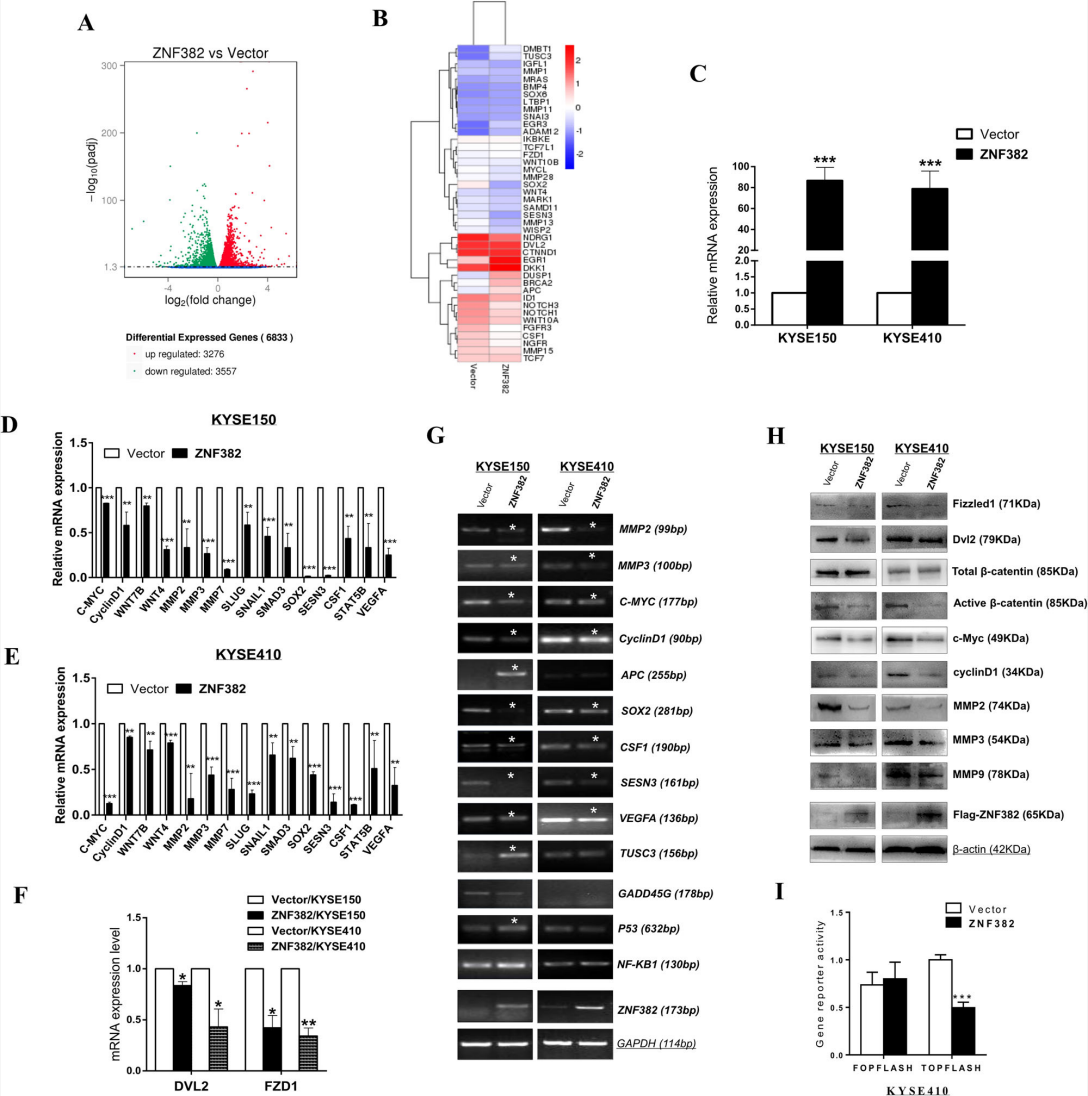
ZNF382 inhibits Wnt/β-catenin signalling activity in ESCC. **a** The entire distribution of differentially expressed genes in *ZNF382*-stably transfected KYSE150 cell is shown by a volcano plot. **b** The hierarchical cluster analysis of selected differentially expressed genes is shown as a heat map. **c** Ectopic expression of *ZNF382* in KYSE150 and KYSE410 cells. Mean ± SD, ***p< 0.001. **d**, **e** Validation of differentially expressed genes in KYSE150 and KYSE410 cells ectopically expressing *ZNF382* by qRT-PCR. Assays were repeated in triplicate. Mean ± SD, ***p< 0.001, **p< 0.01. **f** QRT-PCR was used to evaluate *FZD1* and *DVL2* expression in KYSE150 and KYSE410 cells. Mean ± SD, **p< 0.01, *p< 0.05. Downstream target genes of β-catenin in KYSE150 and KYSE410 cells ectopically expressing *ZNF382* were assayed by RT-PCR (**g**) and western blot (**h**). *GAPDH* and β-actin were used as internal controls, respectively. All assays were performed in triplicate. **i** The effect of *ZNF382* on Wnt/β-catenin signalling was determined by TCF/LEF luciferase reporter activity assays. All the experiments were performed in triplicate. Mean ± SD, ***p< 0.001

To better investigate the underlying mechanism of *ZNF382* in ESCC, RNA-Seq was performed in *ZNF382*-stably transfected KYSE150 cells. The entire distribution of differentially expressed genes was shown by a volcano plot, and a total of 3276 upregulated and 3557 downregulated genes were observed (Fig. 5a, *p < 0.05). We next performed the hierarchical cluster analysis of 42 differentially expressed genes selected on the basis of their expression patterns, and two major groups were classified (Fig. 5b). To further confirm the differential expression of genes identified by RNA-Seq analysis, qRT-PCR was performed to validate the gene expression levels, and 15 differentially expressed genes modulated by *ZNF382* were verified in both KYSE150 and KYSE410 cells (Fig. 5c–e).

Moreover, these significantly regulated genes identified in both KYSE150 and KYSE410 cells were involved in cell metastasis (*SLUG*, *SNAIL* and *SMAD3*), cell growth (*CSF1* and *VEGFA*), stress-induced protector (*SESN3*) and stem cell regulation (*SOX2* and *STAT5B*), adding to our understanding of the possible mechanisms of *ZNF382* tumour suppression in ESCC.

### ZNF382 inhibits Wnt/β-catenin signalling through binding to DVL2 and FZD1 promoters

ChIP-Seq data of HEK293 cells stably expressing eGFP-ZNF382 fusion protein were downloaded from the Gene Expression Omnibus (GEO) database. Enriched peaks located in the transcriptional start sites of *FZD1* and *DVL2* genes were obtained, suggesting that *FZD1* and *DVL2* are the direct targets of *ZNF382* (Supplemental Fig. 3). List of Chip-Seq peaks for *ZNF382* targets were listed in Table S2.

Thus, we hypothesised that *ZNF382* likely plays a role in Wnt/β-catenin signalling through directly binding to *FZD1* and *DVL2* promoters. Reduced *FZD1 and DVL2* accompanied by downstream target genes such as *C-MYC, CCND1, MMP2 and MMP3* expression levels were observed at both the transcriptional (Fig. 5f–g) and protein levels (Fig. 5h). Then, we examined the expression of active β-catenin via western blot. As shown in Figure 5h, the expression of total β-catenin remained unchanged, while the expression of active β-catenin decreased. In addition, inhibition of TOPflash luciferase activity more fully demonstrated that *ZNF382* antagonised Wnt/β-catenin signalling by decreasing active β-catenin levels (Fig. 5i). These results conclusively suggest that *ZNF382* exerts tumour suppression function through antagonising Wnt/β-catenin signalling by directly binding to *DVL2* and *FZD1* promoters.

### Pathway enrichments and protein–protein interaction network

**Fig. 6:**
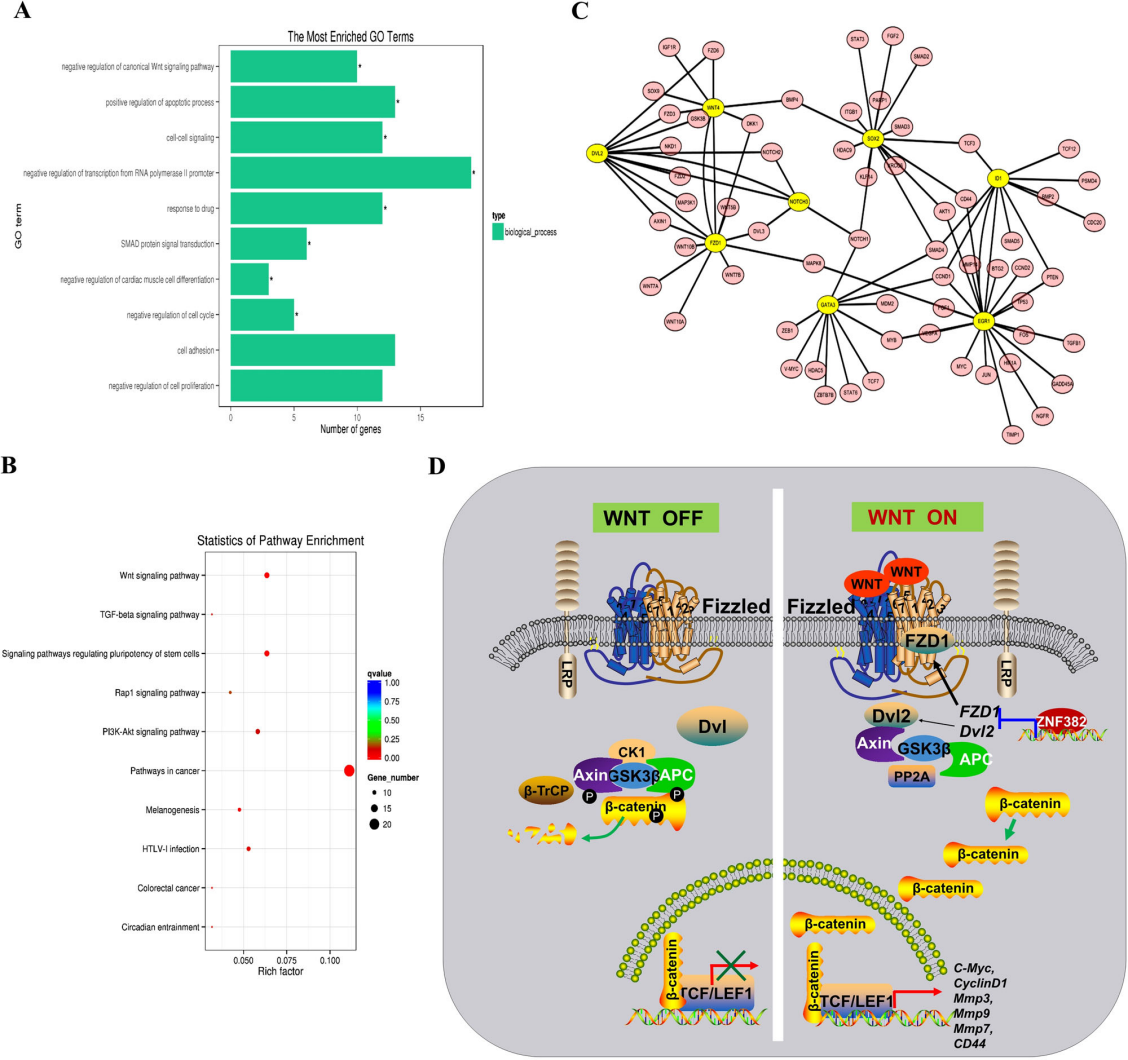
Bioinformatics analysis and possible mechanism of antagonised Wnt/β-catenin signalling by ZNF382 in ESCC. **a** GO biological pathway analysis. * indicates significantly enriched pathways. **b** KEGG pathway categories of differentially expressed genes. The rich factor represents the proportion of differentially expressed genes within a specific term, and the size of the point represents the number of related differentially expressed genes. The *q*-value is the calibrated *p* value. **c** The interactions within the network of genes transcribed by *ZNF382* are shown by the PPI network. Genes in the network are shown as nodes, and edges represent the interactions of genes. **d** A schematic diagram of the mechanism by which *ZNF382* antagonises Wnt/β-catenin signalling in ESCC

Gene Ontology (GO) terms and Kyoto Encyclopedia of Genes and Genomes (KEGG) pathway enrichments based on RNA-Seq data were further analysed and are shown in Figure 6a, b. The Wnt pathway was significantly enriched in both GO and KEGG pathways and other processes, such as apoptosis induction, negative regulation of RNA polymerase II transcription and regulation of stem cell pluripotency were also included. In addition, the protein–protein interaction (PPI) network showed interactions within the network of genes transcribed by *ZNF382*, which involved cell development, cell cycle regulation and metastasis (Fig. 6c).

## Discussion

ZFPs comprising 742 members and involving 423 genes make up the largest transcription factor family in mammals^28,29^. The KRAB domain, located in the amino terminal of ZFP, exerts transcriptional repressor functions^16,19,21,30^ by sequence-specific DNA methylation^29,31,32^. Due to the potent repressor domain, ZFPs play an essential role in cell metabolism, differentiation and apoptosis^21,33,34^. We investigated the novel 19q13 KRAB-ZFP *ZNF382*, which is orthologous to rat *KS1*, and found that *ZNF382* was highly expressed in normal tissues but frequently silenced in multiple carcinomas due to promoter CpG methylation^22,24,29,35^. *ZNF382* was significantly reduced or silenced in ESCC tissues compared with normal oesophageal tissues. The MSP assay also revealed that *ZNF382* promoter was methylated in 87% (99/114) of ESCC cases, suggesting a high frequency of *ZNF382* promoter methylation in ESCC. In addition, data from TCGA database showed that higher level of *ZNF382* heralded a better OS in ESCC patients, indicating that *ZNF382* might be an independent prognostic factor for ESCC patients.

Here, we demonstrated the tumour suppressive effects of *ZNF382* in ESCC cells. Our results indicate that inhibition of cell proliferation by ectopic expression of *ZNF382* in ESCC cells might be through G2/M cell cycle arrest and apoptosis, accompanied by reduced of Cdc25C, Cdc2 and CyclinB1 checkpoint regulators and induced cleavage of apoptotic markers caspase8, caspase3 and PARP. The effect of *ZNF382* on cell metastasis was further evaluated by scratch and transwell assays. The decreased migration and invasion abilities of ESCC cells, with upregulation of epithelial marker CDH1 and downregulation of VIM, CDH2, SNAIL1 and SLUG resulted from negative regulation by *ZNF382*, which was also reflected in the epithelial-mesenchymal transition (EMT) of ESCC cells.

RNA-Seq was additionally performed to explore the underlying mechanism of *ZNF382* in ESCC, and a total of 3276 upregulated and 3557 downregulated genes were identified. Subsequently, 42 differentially expressed genes were selected among which 29 DEGs exhibited a fold change >1.5, for further hierarchical cluster analysis. Validation by qRT-PCR revealed 15 differentially expressed genes modulated by *ZNF382* that were involved in cell metastasis (*SLUG*, *SNAIL* and *SMAD3*), cell growth (*CSF1* and *VEGFA*), stress-induced protection (*SESN3*) and stem cell regulation (*SOX2* and *STAT5B*) were observed in both KYSE150 and KYSE410 cells.

Wnt signalling pathway participates in the carcinogenesis of multiple types of tumours, especially gastrointestinal cancers. Numerous articles have reported that Wnt pathway alterations are involved in the development of oesophageal squamous carcinomas. Wnt signalling mainly includes canonical and noncanonical pathways. The canonical pathway (WnT/β-catenin signalling) is the most frequently reported pathway and has been associated with the regulation of tumour growth, metastasis, and transformation.

In the present investigation, we identified two target genes, *FZD1* and *DVL2*, from ChIP-Seq of HEK293 cells stably transfected with *ZNF382*. Both of these genes are critical elements in the Wnt pathway. *ZNF382* negatively regulates the Wnt/β-catenin signalling by directly binding to *FZD1* and *DVL2* promoters, inhibiting β-catenin activation and T cell factor/lymphoid-enhancer factor (TCF/LEF)-dependent luciferase reporter activity, subsequently downregulating the downstream target genes of β-catenin signalling including *C-MYC, CYCLIND1, MMP2, MMP3* and *MMP9* expressions (Fig. 6d).

It is worth mentioning that amplification of *CYCLIND1*, an essential downstream target gene of β-catenin, has been detected in ESCC samples^1^. Overexpression of *CYCLIND1* is common in ESCC cell lines and is a poor prognostic marker of poor ESCC outcomes, due to its strong association with increased lymph nodes or distant metastasis, high proliferation rates and poor responses to chemotherapy^1^. Thus, evaluation of *CYCLIND1* status is critical for ESCC patients. Here, we found that *ZNF382* negatively modulates Wnt/β-catenin signalling, further inhibiting target gene *CYCLIND1* expression. These findings indirectly illustrate that *ZNF382*, as a TSG, could be a promising prognostic predictor for ESCC patient outcomes.

## Conclusions

In conclusion, *ZNF382* was frequently inactivated by promoter CpG methylation, and suppressed ESCC carcinogenesis through directly regulating Wnt/β-catenin signalling.

## Materials and methods

### Primary tumour samples and cell lines

Multiple ESCC cell lines including KYSE150, KYSE410 and KYSE510 were used^24^. The cells were cultured in RPMI 1640 medium (Gibco-BRL, Karlsruhe, Germany), supplemented with 10% foetal bovine serum (Gibco-BRL), at 37 °C in 5% CO_2_ atmosphere. Primary ESCC tissues, adjacent non-cancerous tissues and normal oesophageal tissues were obtained from patients who underwent surgery at the Department of Cardiothoracic Surgery of the First Affiliated Hospital of Chongqing Medical University. All samples were stored at −80 °C until being evaluated by pathologists at the Chongqing Key Laboratory of Molecular Oncology and Epigenetic of the First Affiliated Hospital of Chongqing Medical University. All participants provided written consent before enrolment, and the study was approved by the Ethics Committees of the First Affiliated Hospital of Chongqing Medical University.

### RNA and DNA extraction

Total RNA was isolated from cells and tissues with the TRIzol reagent (Invitrogen, Carlsbad, CA, USA). Genomic DNA was extracted from cells and tissues with a QIAamp DNA Mini Kit (Qiagen, Hilden, Germany) following the manufacturers’ protocols^36^. RNA and DNA concentrations were measured with a NanoDrop 2000 spectrophotometer (Thermo Fisher Scientific, Waltham, MA), and sample quality was determined by gel electrophoresis.

### Reverse transcription, semi-quantitative PCR (RT-PCR) and quantitative PCR (qRT-PCR)

Reverse transcription and semi-quantitative PCR (RT-PCR) were performed as previously described using Go-Taq polymerase (Promega, Madison, WI)^37^. RT-PCR was performed using a final volume of 10 μL reaction mixture containing 2 μL cDNA. *GAPDH* served as an internal control. The amplified PCR products were assayed on 2% agarose gels. A SYBR Green PCR Master Mix kit (Invitrogen) and an Applied Biosystems 7500 Real-Time PCR System (Applied Biosystems, Foster City, CA) were used for qRT-PCR^36^. The relative expression of *ZNF382* was estimated with the 2(−ΔCt) method^38^ and all assays were performed in triplicate. The primers used are listed in Supplementary Table 1.

### 5-Aza-2′-deoxycytidine (Aza) and trichostatin A (TSA) treatment

Combined treatment of Aza, a DNA methyltransferase inhibitor and TSA, a HDAC inhibitor synergistically activated methylated genes. ESCC cell lines were treated with 10 mmol/L Aza (Sigma-Aldrich, Steinheim, Germany) for three days and 100 nmol/L TSA (Sigma-Aldrich) for an additional 24 h as previously described^24^. Cells were then used for RNA and DNA isolation.

### Bisulfite treatment and methylation-specific PCR (MSP)

To evaluate the methylation status of *ZNF382*, bisulfite modification of DNA and MSP were performed as previously described^24,39,40^. In brief, bisulfite-treated DNA was amplified by MSP with methylation-specific primers. Methylated and non-methylated DNAs were used as positive and negative controls. MSP products were separated on 2% agarose gels (MBI Fermentas, Vilnius, Lithuania). The primers are listed in Supplementary Table 1.

### Transfection and construction of stable cell lines

A *ZNF382* expression vector (PcDNA3.1–*ZNF382*-FLAG) and a control vector (PcDNA3.1) were used^24^. Plasmids were transfected into ESCC cell lines using Lipofectamine 2000 (Invitrogen) at a final concentration of 4 μg. The transfected cells were harvested at 48 h. *ZNF382*-stably transfected ESCC cells were selected with G418 (Invitrogen, Gibco) for 2 weeks.

### Knockdown of ZNF382

*ZNF382* siRNA kits were purchased from GenePharma (Jiangsu, China). All transfections were performed using Lipofectamine 2000 (Invitrogen) according to the manufacturer’s instruction with a concentration of 50 nM siRNA. The cells were harvested for subsequent assays at 72 h after transfection.

### Cell viability assay

Stably transfected KYSE150, KYSE410 and KYSE510 cells were seeded in 96-well plates and grown overnight. Cell viabilities were then evaluated with a Cell Counting Kit-8 (Beyotime Institute of Biotechnology, Jiangsu, China) at 24, 48 and 72 h, respectively. All experiments were independently repeated three times.

### Colony formation assay

Stably transfected KYSE150, KYSE410 and KYSE510 cells were plated in six-well plates and cultured with various concentrations of G418 for 10–14 days. Surviving colonies with >50 cells were counted following fixation and stained with gentian violet (Beyotime). All experiments were repeated three times.

### 5-Ethynyl-2′-deoxyuridine (Edu) cell proliferation assay

Cell proliferation was evaluated with an Edu cell proliferation kit (RiboBio, Guangzhou, China). Edu is a thymine nucleoside analogue that replaces thymine during DNA replication, and DNA replication activity is assayed by labelling with specific Edu and Apollo fluorescent dyes. All assays were repeated three times.

### Wound healing and transwell assay

Cell mobility was evaluated with a wound healing assay. Briefly, *ZNF382*-stably transfected KYSE150, KYSE410 and KYSE510 cells were seeded in six-well plates and grown until confluence. Linear scratch wounds were created with a pipette tip and cell migration distance was measured at various times by phase contrast microscopy (Leica DMI4000B, Milton Keynes, Bucks, UK).

Transwell chambers with an 8 μm pores (Corning Life Sciences, Bedford, MA) were used with or without Matrigel (BD Biosciences, San Jose, CA) or not to measure cell migration and invasion, respectively. Cells on the lower membrane surface were counted following fixation and staining. All assays were performed in triplicate.

### Flow cytometry assay

Cell cycle arrest and apoptosis were assayed via flow cytometry as previously described^37^. In brief, the cells were harvested with trypsin, fixed with ice-cold 70% ethanol and stained with propidium iodide (PI) to assay the cell cycle distribution. Apoptosis was assayed with annexin V–fluorescein isothiocyanate and PI following the kit manufacturer’s protocol. All data were measured using a Cell Quest kit (BD Biosciences) and experiments were independently repeated in triplicate.

### Western blot assay

Western blot assays were performed as previously described^36^. Briefly, whole cells lysates were prepared with lysis buffer (Beyotime) containing a protease inhibitor cocktail (Sigma-Aldrich, St. Louis, MO). Lysates were sonicated, and the protein concentration was determined using a bicinchoninic acid (BCA) assay. Aliquots of 50 µg protein were separated with 10–12% sodium dodecyl sulphate polyacrylamide gel electrophoresis and then transferred to polyvinylidene fluoride membranes (Merck Millipore, Billerica, MA). After blocking with 5% nonfat milk, the membranes were incubated at 4 °C overnight with anti-DDK (FLAG) monoclonal antibody (TA50011-100; OriGene, Rockville, MD), anti-FLAG M2 antibody (#14793; Cell Signaling Technology, Danvers, MA), cleaved caspase 3 (#9661, Cell Signaling Technology), cleaved caspase 8 (#8592; Cell Signaling Technology), cleaved PARP (#5625, Cell Signaling Technology), cyclin D1 (#1677; Epitomics, Burlingame, CA), c-Myc (#1472-1, Epitomics), active β-catenin (#05-665,Merck Millipore), total β-catenin (#2677, Cell Signaling Technology), β-actin (sc-47778; Santa Cruz Biotechnology), E-cadherin (#1702-1, Epitomics), vimentin (#2707-1, Epitomics), N-cadherin (ab98952; Abcam), Snail1 (#3897;Cell Signaling Technology), SLUG (#9585, Cell Signaling Technology), anti-Frizzled homologue 1 antibody (ab71342, Abcam), Dvl2 (sc-420081, Santa Cruz), Cdc2 (sc-8395, Santa Cruz), Cdc25C (sc-24540, Santa Cruz), or cyclinB1 (sc-4073, Santa Cruz) primary monoclonal antibodies. Membranes were visualised with Electrochemiluminescence Plus Detection Reagents (RPN2132; GE Healthcare Life Science, Buckinghamshire, UK). All assays were independently repeated three times.

### Immunohistochemistry (IHC)

IHC was performed with an UltraSensitive SP Kit (Maixin-Bio, Fujian, China) following the manufacturer’s instructions as previously described^41,42^. Cells were incubated with a primary antibody against *ZNF382* (1:50 dilution, Sigma-ldrich) overnight at 4 °C and then incubated with a secondary antibody at 37 °C for 30 min. All IHC images were captured under a microscope at ×400 magnification.

### Dual-luciferase reporter assay

Briefly, cells were co-transfected with pcDNA3.1–*ZNF382*-FLAG or pcDNA3.1 and TCF-responsive luciferase construct TOPflash or FOPflash reporter plasmids plus Renilla luciferase reporter pRL-TK in 24-well plates. Luciferase activities were read 48 h later using a dual-luciferase reporter assay kit (Promega). All assays were independently performed three times.

### RNA sequencing (RNA-Seq)

RNA-Seq was performed by the Novogene Bioinformatics Technology Company (Beijing, China). RNA integrity was assessed with an RNA Nano 6000 Assay Kit included with the Bioanalyzer 2100 system (Agilent Technologies, CA). Sequencing libraries were prepared using a NEBNext Ultra RNA LibraryPrep Kit for Illumina (New England Biolabs) following the manufacturer’s recommendations. RNA-Seq was performed with an Illumina Hiseq 2000/2500 platform. Bowtie v. 2.0.6^43–45^ and TopHat v. 2.0.9^46–48^ were used for reads mapping to the reference genome. Genes with an adjusted *p* or0.05 and identified with DESeq^49,50^ were considered to be differentially expressed.

### Peaks calling for chromatin immunoprecipitation sequencing (ChIP-Seq)

ChIP-Seq of ZNF382 from human HEK293 cells were obtained from the Gene Expression Omnibus (GEO, https://www.ncbi.nlm.nih.gov/geo) database. The GSM2424064_ENCFF191YCR_fold_change_over_control_GRCh38.bigWig file was downloaded. We annotated those peaks by using R package “CHIPseeker”^51^. Subsequently, the gene region were intercepted with BEDTools^52^ and exported to Integrated Genomics Viewer software to map the peaks^53,54^.

### Pathway enrichment analysis

The function enrichments of differentially expressed genes were analysed with GO classification^55^ and Kyoto Encyclopedia of Genes and Genomes (KEGG) pathways^56^ from the Database for Annotation, Visualization and Integrated Discovery (DAVID, http://david.abcc.ncifcrf.gov/)^57^. The level of significance was *p<* 0.05.

### PPI analysis

The PPI analysis of target genes was based on the STRING database (https://string-db.org/) and the networks were constructed by Cytoscape^58^.

### Statistical analysis

Data were reported as the means ± standard deviation (SD). The significance of differences between the experimental and control values were tested the with two-tailed Student's *t*-test. Correlations of methylation status and clinicopathological features were evaluated using the χ2 and Fisher’s exact tests. For all tests, *p<* 0.05 was considered statistically significant.

### Availability of data and materials

Data of prognostic significance is available from Cancer Genome Atlas database (http://www.cbioportal.org/). CHIP-Seq data analysed during the present study is available from Gene Expression Omnibus database (GEO, https://www.ncbi.nlm.nih.gov/geo). PPI analysis is available from the STRING database (https://string-db.org/). Schematic model drafted with the tool scienceslides software is available at http://www.scienceslides.com/.

## Electronic supplementary material

Supplemental figures

List of primers used in this study

List of CHIP-Seq peaks for ZNF382 targets

Supplementary figure legends
