# Supplementary figures and images for: The novel 19q13 KRAB zinc-finger tumour suppressor ZNF382 is frequently methylated in oesophageal squamous cell carcinoma and antagonises Wnt/β-catenin signalling
Source: Cell Death Dis. 2018 May 14;9(5):573. doi: 10.1038/s41419-018-0604-z (PMC5951945; doi:10.1038/s41419-018-0604-z)

**Supplementary Figures**

**Figure S1**

**
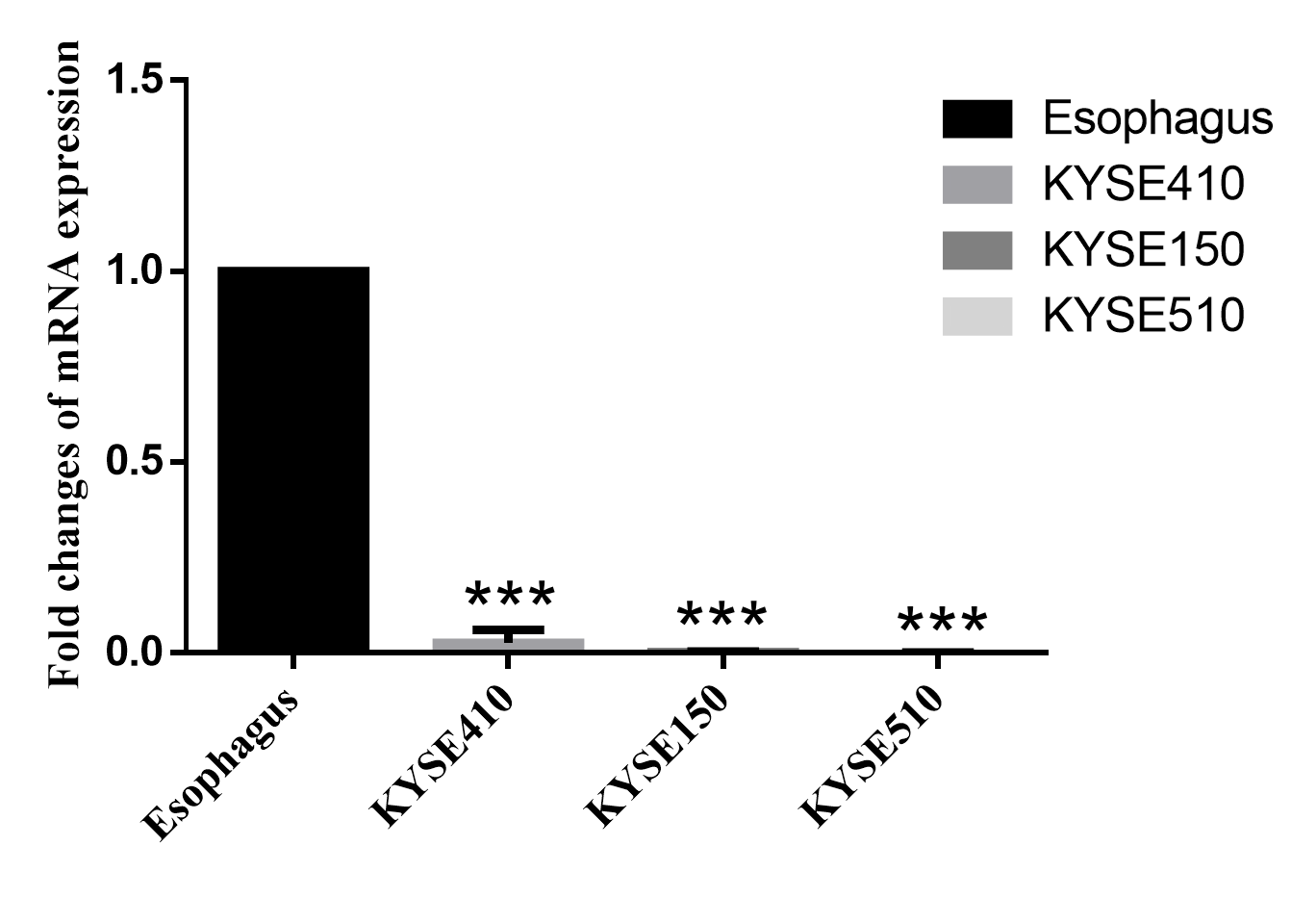
**

**Figure S2**

**
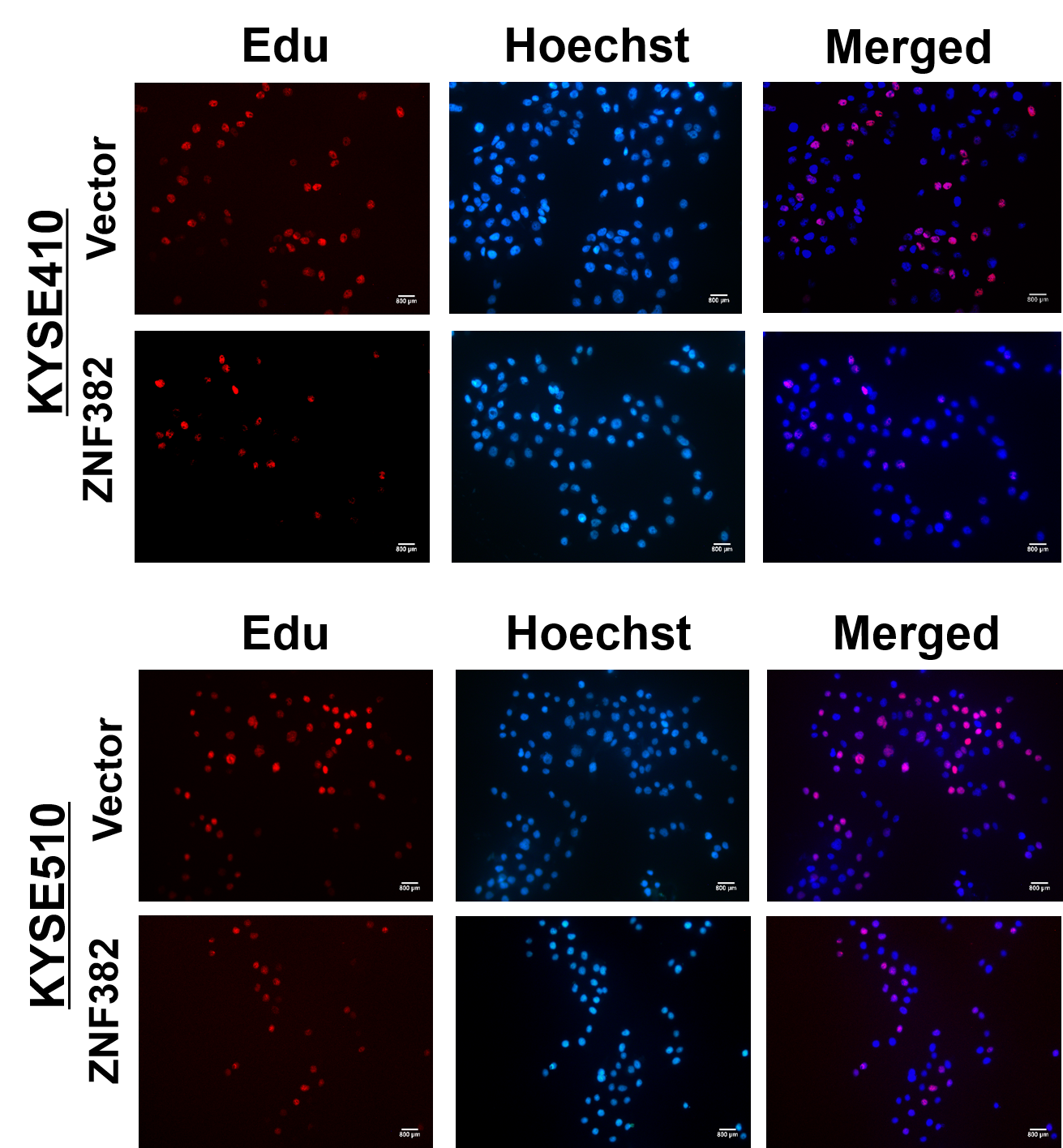
**

**Figure S3**

**
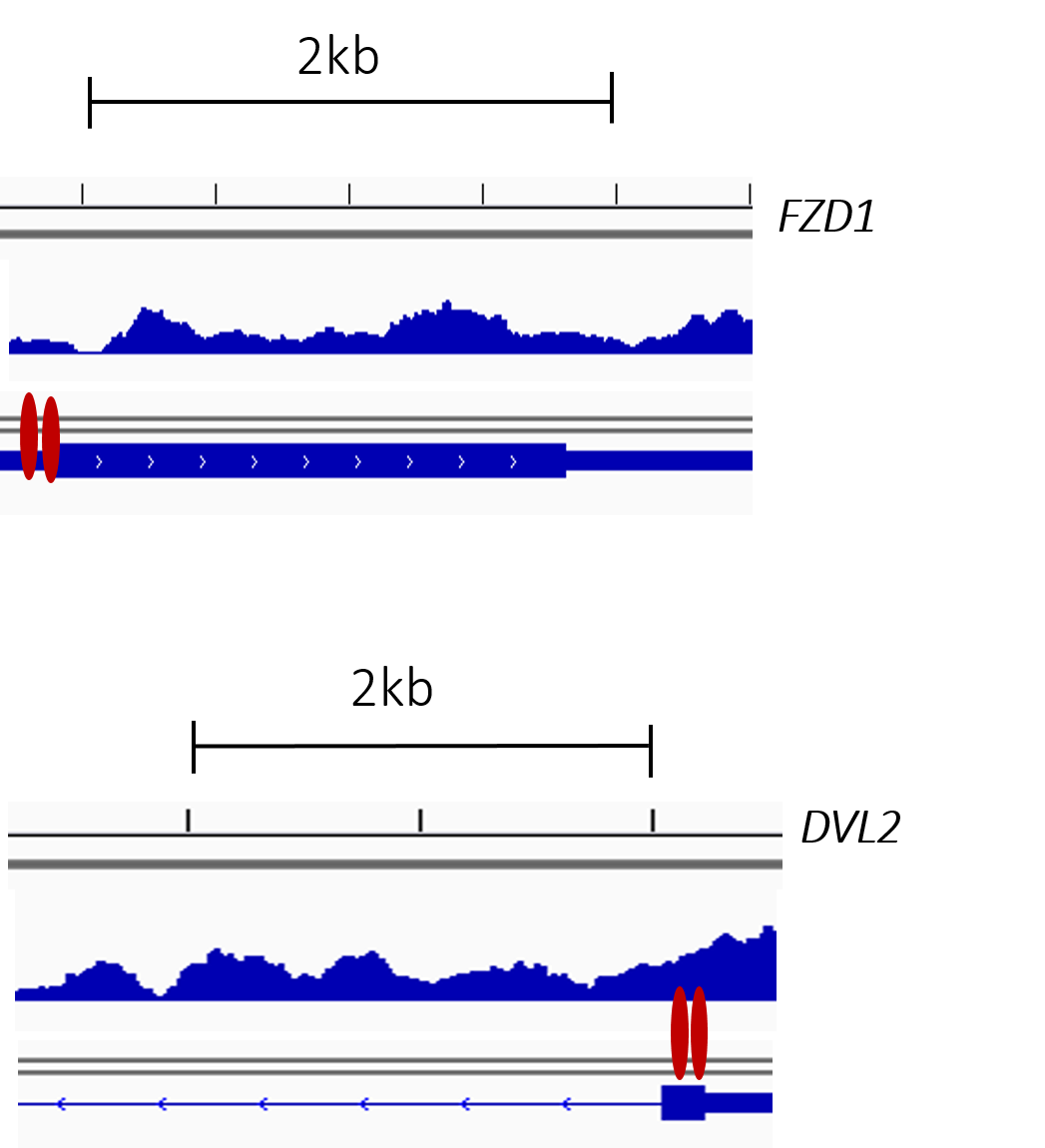
**

Supplement: Supplementary file 1 — Supplemental figures [file 41419_2018_604_MOESM1_ESM.doc]
