# Supplementary material for: The novel 19q13 KRAB zinc-finger tumour suppressor ZNF382 is frequently methylated in oesophageal squamous cell carcinoma and antagonises Wnt/β-catenin signalling
Source: Cell Death Dis. 2018 May 14;9(5):573. doi: 10.1038/s41419-018-0604-z (PMC5951945; doi:10.1038/s41419-018-0604-z)
Supplement: Supplementary file 2 — List of primers used in this study [file 41419_2018_604_MOESM2_ESM.docx]

Table S1. List of primers used in this study

| **PCR** | **Primer** | **Sequence (5'-3')** | **Product size (bp)** | **PCR Cycles** | **Annealing temperature (℃)** |
| --- | --- | --- | --- | --- | --- |
| **RT-**  **PCR**  **qRT-**  **PCR**  **MSP** | *ZNF382F* | CCTTACAGGGATCAGTGTCA | **173bp** | **32** | 55 |
|  | *ZNF382R* | CAACTTGCGATCATATCAG |  |  |  |
|  | *GAPDHF* | CCAGCAAGAGCACAAGAGGAA | **114bp** | **23** | 55 |
|  | *GAPDHR* | GGTCTACATGGCAACTCAAGG |  |  |  |
|  | *CyclinD1F* | CTAGCAAGCTGCCGAACC | **90bp** |  | **60** |
|  | *CyclinD1R* | TCCGAGCACAGGATGACC |  |  |  |
|  | *C-MycF* | GGAGGCTATTCTGCCCATTT | **177bp** |  | 60 |
|  | *C-MycR* | GTCGAGGTCATAGTTCCTGTTGG |  |  |  |
|  | *SESN3F* | ATACTCTACATCCGGTCGTCTG | **161bp** |  | **60** |
|  | *SESN3R* | GTCTAGCTGCAGCCATTATTGC |  |  |  |
|  | *CSF1F* | TGAGGCTGAAGAGCTGCTTC | **190bp** |  | **60** |
|  | *CSF1R* | CTGGAGCATTCAGCAAAGCTGT |  |  |  |
|  | *FZD1F* | GCGACGTACTGAGCGGAGTG | **150bp** |  | **60** |
|  | *FZD1R* | TGATGGTGCGGATGCGGAAG |  |  |  |
|  | *DVL2F* | GACGAAGGTGATTTACCACC | **194bp** |  | **60** |
|  | *DVL2R* | GGCGGGCGTTGTCATCTG |  |  |  |
|  | *Wnt7BF* | TTTGGCGTCCTCTACGTGAAG | **145bp** |  | **60** |
|  | *Wnt7BR* | CCCCGATCACAATGATGGCA |  |  |  |
|  | *Wnt4F* | TGTGGCCTTCTCACAGTCGTT | **180bp** |  | **60** |
|  | *Wnt4R* | CGCCAGCACGTCTTTACCTC |  |  |  |
|  | *STAT5BF* | GATCAAGCTGGGGCACTATG | **161bp** |  | **60** |
|  | *STAT5BR* | ACATGGCATCAGCAAGGCTT |  |  |  |
|  | *MMP2F* | CATACAGGATCATTGGCTACACAC | **99bp** |  | 60 |
|  | *MMP2R* | GCAGTGGGGTCACATCGCT |  |  |  |
|  | *MMP3F* | CTACAAGGAGGCAGGCAAGAC | **100bp** |  | 60 |
|  | *MMP3R* | ACGCACAGCAACAGTAGGAT |  |  |  |
|  | *SNAILF* | GAGGCGGTGGCAGACTAG | **159bp** |  | 60 |
|  | *SNAILR* | GACACATCGGTCAGACCAG |  |  |  |
|  | *MMP7F* | AGATGCTCACTTCGATGAGG | **130bp** |  | 60 |
|  | *MMP7R* | CACTGCATTAGGATCAGAGG |  |  |  |
|  | *VEGFAF* | CACACAGGATGGCTTGAAGA | **136bp** |  | 60 |
|  | *VEGFAR* | AGGGCAGAATCATCACGAAG |  |  |  |
|  | *ZNF382m1* | GGCGATTAACGGGTCGTTTC | **230bp** |  | 60 |
|  | *ZNF382m2* | AAAATTTCCAAACCCGACTCG |  |  |  |
|  | *ZNF382u1* | GTGGTGATTAATGGGTTGTTTT | **233bp** |  | 58 |
|  | *ZNF382u2* | CAAAATTTCCAAACCCAACTCA |  |  |  |
