# Supplementary material for: The novel 19q13 KRAB zinc-finger tumour suppressor ZNF382 is frequently methylated in oesophageal squamous cell carcinoma and antagonises Wnt/β-catenin signalling
Source: Cell Death Dis. 2018 May 14;9(5):573. doi: 10.1038/s41419-018-0604-z (PMC5951945; doi:10.1038/s41419-018-0604-z)
Supplement: Supplementary file 3 — List of CHIP-Seq peaks for ZNF382 targets [file 41419_2018_604_MOESM3_ESM.doc]

**List of CHIP-Seq peaks for ZNF382 targets**

| Fold enrichment | Seq  names | Start | End | Closest TSS Gene symbol | Gene Description |
| --- | --- | --- | --- | --- | --- |
| 430.4971536 | chr14 | 62161739 | 62162167 | HIF1A | Hypoxia inducible factor 1 alpha subunit |
| 182.5906 | chr19 | 49457819 | 49458120 | BAX | BCL2 associated X |
| 128.5771151 | chr17 | 40540567 | 40541051 | STAT3 | Signal transducer and activator of transcription 3 |
| 135.9623517 | chr4 | 185571252 | 185571429 | CASP3 | Caspase 3 |
| 127.8855617 | chr7 | 142985022 | 142985200 | CASP2 | Caspase 2 |
| 109.5495216 | chr7 | 90893488 | 90893972 | FZD1 | Frizzled class receptor 1 |
| 109.7350734 | chr1 | 15853093 | 15853577 | CASP9 | Caspase 9 |
| 92.33417729 | chr19 | 30301992 | 30302476 | CCNE1 | Cyclin E1 |
| 76.10128 | chr19 | 12902094 | 12902578 | JUNB | JunB proto-oncogene |
| 74.7070341 | chr19 | 40791508 | 40791992 | AKT2 | AKT serine/threonine kinase 2 |
| 65.36109229 | chr2 | 202096843 | 202097327 | CASP8 | Caspase 8 |
| 65.24897049 | chr6 | 109416295 | 109416779 | SESN1 | Sestrin 1 |
| 64.60625319 | chr18 | 45457630 | 45458114 | SMAD2 | SMAD family member 2 |
| 59.98148396 | chr19 | 54023851 | 54024335 | ZNF331 | Zinc finger protein 331 |
| 57.70423545 | chrX | 47441584 | 47442068 | TIMP1 | TIMP metallopeptidase inhibitor 1 |
| 54.8337898 | chr19 | 42747037 | 42747521 | GSK3A | Glycogen synthase kinase 3 alpha |
| 54.26529 | chr20 | 30191837 | 30192321 | ID1 | Inhibitor of DNA binding 1 |
| 51.65641 | chr14 | 75745347 | 75745831 | FOS | Fos proto-oncogene |
| 51.02068579 | chr6 | 21593472 | 21593956 | SOX4 | SRY-box 4 |
| 50.50555296 | chr19 | 1652874 | 1653358 | TCF3 | Transcription factor 3 |
| 49.74282882 | chr13 | 26828002 | 26828486 | CDK8 | Cyclin dependent kinase 8 |
| 49.37931 | chr19 | 45982189 | 45982673 | ERCC1 | ERCC excision repair 1 |
| 47.94495681 | chr12 | 56753742 | 56754226 | STAT2 | Signal transducer and activator of transcription 2 |
| 47.3592425 | chr5 | 142077472 | 142077956 | FGF1 | Fibroblast growth factor 1 |
| 47.29662352 | chr3 | 119814132 | 119814616 | GSK3B | Glycogen synthase kinase 3 beta |
| 46.65134711 | chr19 | 45970977 | 45971461 | FOSB | FosB proto-oncogene |
| 46.47866731 | chr19 | 2476053 | 2476537 | GADD45B | Growth arrest and DNA damage inducible beta |
| 46.41949314 | chr5 | 112043184 | 112043668 | APC | APC |
| 46.04088432 | chr19 | 14202183 | 14202667 | SAMD1 | Sterile alpha motif domain containing 1 |
| 45.73119687 | chr16 | 14013262 | 14013746 | ERCC4 | ERCC excision repair 4 |
| 45.28356638 | chr12 | 68726317 | 68726801 | MDM1 | Mdm1 nuclear protein |
| 45.22796572 | chr1 | 226595762 | 226596246 | PARP1 | Poly(ADP-ribose) polymerase 1 |
| 44.19120224 | chr12 | 24103642 | 24104126 | SOX5 | SRY-box 5 |
| 44.0993679 | chr1 | 59250642 | 59251126 | JUN | Jun proto-oncogene |
| 44.00914869 | chr12 | 69201359 | 69201843 | MDM2 | MDM2 proto-oncogene |
| 43.30319085 | chr1 | 244014412 | 244014896 | AKT3 | AKT serine/threonine kinase 3 |
| 43.19576527 | chr2 | 85360105 | 85360589 | TCF7L1 | Transcription factor 7 like 1 |
| 43.03468397 | chr12 | 56360522 | 56361006 | CDK2 | Cyclin dependent kinase 2 |
| 42.87258981 | chr1 | 120611896 | 120612380 | NOTCH2 | Notch 2 |
| 42.785779 | chr15 | 57210521 | 57211005 | TCF12 | Transcription factor 12 |
| 42.67037674 | chr16 | 30134531 | 30135015 | MAPK3 | Mitogen-activated protein kinase 3 |
| 42.66256435 | chr12 | 92539808 | 92540292 | BTG1 | Anti-proliferation factor 1 |
| 41.07804142 | chr12 | 102874883 | 102875367 | IGF1 | Insulin like growth factor 1 |
| 40.59872523 | chr20 | 61493423 | 61493907 | TCFL5 | Transcription factor like 5 |
| 40.28918857 | chr6 | 43737257 | 43737741 | VEGFA | Vascular endothelial growth factor A |
| 40.00813463 | chr15 | 66994472 | 66994956 | SMAD6 | SMAD family member 6 |
| 39.81478356 | chr17 | 7137511 | 7137995 | DVL2 | Dishevelled segment polarity protein 2 |
| 39.25343263 | chr1 | 203274483 | 203274967 | BTG2 | BTG anti-proliferation factor 2 |
| 38.84253156 | chr1 | 40367693 | 40368177 | MYCL | V-myc avian myelocytomatosis Viral  Oncogene Homolog 1 |
| 38.48804268 | chr8 | 28351325 | 28351809 | FZD3 | Frizzled class receptor 3 |
| 37.67872252 | chr19 | 18392743 | 18393227 | JUND | JunD proto-oncogene |
| 37.35382556 | chr19 | 45250588 | 45251072 | BCL3 | B-cell CLL/Lymphoma 3 |
| 35.86386886 | chr11 | 64051726 | 64052210 | BAD | BCL2 associated agonist of cell death |
| 35.55957535 | chr7 | 152372912 | 152373396 | XRCC2 | X-ray repair cross complementing 2 |
| 34.22853004 | chr15 | 59397188 | 59397672 | CCNB2 | Cyclin B2 |
| 33.86410505 | chr5 | 68462785 | 68463269 | CCNB1 | Cyclin B1 |
| 33.77625367 | chr8 | 22551003 | 22551487 | EGR3 | Early growth response 3 |
| 33.4995151 | chr11 | 70048896 | 70049380 | FADD | Fas associated via death domain |
| 31.35178426 | chr10 | 31607297 | 31607781 | ZEB1 | Zinc finger E-box binding homeobox 1 |
| 31.33344686 | chr16 | 403112 | 403596 | AXIN1 | Axin 1 |
| 30.88883428 | chr16 | 58059189 | 58059673 | MMP15 | Matrix metallopeptidase 15 |
| 30.57693567 | chr18 | 25757115 | 25757599 | CDH2 | Cadherin 2 |
| 30.38867074 | chr17 | 7590537 | 7591021 | TP53 | Tumor protein p53 |
| 30.09683614 | chr20 | 48599444 | 48599928 | SNAI1 | Snail family transcriptional repressor 1 |
| 30.07186464 | chr8 | 120885783 | 120886267 | DEPTOR | DEP domain containing mtor-interacting protein |
| 29.9959 | chr18 | 60987667 | 60988151 | BCL2 | BCL2 |
| 29.46623795 | chr8 | 128747476 | 128747960 | MYC | C-Myc |
| 29.33178366 | chr6 | 147828998 | 147829482 | SAMD5 | Sterile alpha motif domain containing 5 |
| 29.22839432 | chr13 | 22244896 | 22245380 | FGF9 | Fibroblast growth factor 9 |
| 28.55991391 | chr11 | 94965454 | 94965938 | SESN3 | Sestrin 3 |
| 27.55381121 | chr1 | 154377519 | 154378003 | IL6R | Interleukin 6 receptor |
| 27.25837898 | chr22 | 41487633 | 41488117 | EP300 | E1A binding protein p300 |
| 27.06481412 | chr19 | 47165272 | 47165756 | DACT3 | Dishevelled binding antagonist of beta catenin 3 |
| 26.42938967 | chr20 | 62610930 | 62611414 | SAMD10 | Sterile alpha motif domain containing 10 |
| 26.16383991 | chr10 | 35930902 | 35931386 | FZD8 | Frizzled class receptor 8 |
| 26.09658661 | chr8 | 38325999 | 38326483 | FGFR1 | Fibroblast growth factor receptor 1 |
