# Supplementary material for: The novel 19q13 KRAB zinc-finger tumour suppressor ZNF382 is frequently methylated in oesophageal squamous cell carcinoma and antagonises Wnt/β-catenin signalling
Source: Cell Death Dis. 2018 May 14;9(5):573. doi: 10.1038/s41419-018-0604-z (PMC5951945; doi:10.1038/s41419-018-0604-z)
Supplement: Supplementary file 4 — Supplementary figure legends [file 41419_2018_604_MOESM4_ESM.docx]

***Supplemental data***

***Figure S1.*** *ZNF382* expression in ESCC cells compared with in the normal oesophagus tissues by qRT-PCR. Assays were repeated in triplicate. Mean ± SD, ****p* < 0.001

***Figure S2.*** Edu incorporation assay and images of KYSE410 and KYSE510 cells after ectopically expressing of *ZNF382* are shown.

***Figure S3.*** ZNF382 binding peaks located in *FZD1* and *DVL2* gene promoters were mapped using the Integrative Genomics Viewer software.

***Table S1.*** List of primers used in this study.

***Table S2.*** List of CHIP-Seq peaks for *ZNF382* targets.
